# Longitudinal Methods in Orthognathic Surgery Studies

**DOI:** 10.1186/s40902-025-00496-3

**Published:** 2025-12-30

**Authors:** Mehrdad Farrokhi, Mohammad Amin Nochian

**Affiliations:** 1 Eris Research Institute, Tehran, Islamic Republic of Iran; 2 Student Research Committee, Faculty of Nursing and Midwifery, Semnan University of Medical Sciences, Semnan, Islamic Republic of Iran

**Keywords:** Orthognathic surgery, Soft tissue canting, Repeated measures analysis

## Abstract

This letter provides a methodological comment on the recent study by Kim et al., which evaluated soft tissue canting at preoperative, postoperative, and post-debonding stages in the same group of patients. The correspondence highlights that these measurements represent dependent observations and therefore require appropriate repeated-measures statistical methods. It emphasizes that one-way analysis of variance is designed for independent groups and is not suitable for longitudinal data. The aim of this letter is to encourage accurate statistical practice in studies involving repeated measurements in orthognathic and orthodontic research.

## To the editor

We read with great interest the recently published article by Kim et al. [1] in the journal of Maxillofacial Plastic and Reconstructive Surgery. This study aimed to evaluate soft tissue canting parameters including eye canting, lip canting, soft tissue chin deviation, and commissure height difference at three distinct stages of treatment in patients undergoing bimaxillary orthognathic surgery followed by orthodontic treatment. These parameters were measured using standardized frontal photographs and cephalometric radiographs at the following time points. (1) preoperative (T0), (2) postoperative (T1), and (3) post debonding (T2). The authors compared these measurements within the same sample of 25 patients. Because all comparisons were performed within a single group of patients at different time points of assessment, the repeated observations were dependent on each other (as shown in Figs. 2, 3 and 4). However, as stated in the abstract and in the statistical analysis section of the methods, one way analysis of variance ANOVA was used to compare quantitative variables across the three stages of measurement. One way ANOVA is generally appropriate for comparing normally distributed quantitative variables between more than two independent groups [2, 3]. Since the comparisons in this study involved repeated measurements collected from the same group of patients across T0, T1, and T2, the authors should first assess the normal distribution of the quantitative variables and then apply repeated Measures ANOVA for normally distributed data or the Friedman test when normal distribution is not satisfied [4, 5].

## Data Availability

No datasets were generated or analysed during the current study.
